# Clinical Impact of Monoclonal Antibodies in the Treatment of High-Risk Patients with SARS-CoV-2 Breakthrough Infections: The ORCHESTRA Prospective Cohort Study

**DOI:** 10.3390/biomedicines10092063

**Published:** 2022-08-24

**Authors:** Alessia Savoldi, Matteo Morra, Alessandro Castelli, Massimo Mirandola, Matilda Berkell, Mathias Smet, Angelina Konnova, Elisa Rossi, Salvatore Cataudella, Pasquale De Nardo, Elisa Gentilotti, Akshita Gupta, Daniele Fasan, Enrico Gibbin, Filippo Cioli Puviani, Jan Hasenauer, Roy Gusinow, Adriana Tami, Samir Kumar-Singh, Surbhi Malhotra-Kumar, mAb ORCHESTRA Working Group, Evelina Tacconelli

**Affiliations:** 1 Division of Infectious Diseases, Department of Diagnostics and Public Health, University of Verona, P.le L.A. Scuro 10, 37134 Verona, Italy; 2 School of Health Sciences, University of Brighton, Brighton BN2 4AT, UK; 3 Lab of Medical Microbiology, Vaccine & Infectious Disease Institute, University of Antwerp, 2610 Antwerp, Belgium; 4 Molecular Pathology Group, Cell Biology & Histology, Faculty of Medicine and Health Sciences, University of Antwerp, 2610 Antwerp, Belgium; 5 CINECA—Interuniversity Consortium, Via Magnanelli 6/3, Casalecchio di Reno, 40033 Bologna, Italy; 6 Helmholtz Center Munich—Germany Research Center for Environmental Heath, Institute for Computational Biology, 85764 Neuherberg, Germany; 7 Life and Medical Sciences Institute, University of Bonn, 53115 Bonn, Germany; 8 Department of Medical Microbiology & Infection Prevention, University Medical Center Groningen, 9713 AV Groningen, The Netherlands

**Keywords:** anti-spike monoclonal antibodies, COVID-19 breakthrough infection, COVID-19 early treatment

## Abstract

The clinical impact of anti-spike monoclonal antibodies (mAb) in Coronavirus Disease 2019 (COVID-19) breakthrough infections is unclear. We present the results of an observational prospective cohort study assessing and comparing COVID-19 progression in high-risk outpatients receiving mAb according to primary or breakthrough infection. Clinical, serological and virological predictors associated with 28-day COVID-19-related hospitalization were identified using multivariate logistic regression and summarized with odds ratio (aOR) and 95% confidence interval (CI). A total of 847 COVID-19 outpatients were included: 414 with primary and 433 with breakthrough infection. Hospitalization was observed in 42/414 (10.1%) patients with primary and 8/433 (1.8%) patients with breakthrough infection (*p* < 0.001). aOR for hospitalization was significantly lower for breakthrough infection (aOR 0.12, 95%CI: 0.05–0.27, *p* < 0.001) and higher for immunocompromised status (aOR:2.35, 95%CI:1.08–5.08, *p* = 0.003), advanced age (aOR:1.06, 95%CI: 1.03–1.08, *p* < 0.001), and male gender (aOR:1.97, 95%CI: 1.04–3.73, *p* = 0.037). Among the breakthrough infection group, the median SARS-CoV-2 anti-spike IgGs was lower (*p* < 0.001) in immunocompromised and elderly patients >75 years compared with that in the immunocompetent patients. Our findings suggest that, among mAb patients, those with breakthrough infection have significantly lower hospitalization risk compared with patients with primary infection. Prognostic algorithms combining clinical and immune-virological characteristics are needed to ensure appropriate and up-to-date clinical protocols targeting high-risk categories.

## 1. Introduction

Severe Acute Respiratory Syndrome Coronavirus 2 (SARS-CoV-2) vaccination has become the key strategy to reduce the Coronavirus disease 2019 (COVID-19) related hospitalization and relieve the burden on the healthcare system. Vaccination campaigns are at an advanced stage in several countries; however, despite the high efficacy of the Food and Drug Administration (FDA) and European Medicine Agency (EMA) authorized vaccinations, some concerns have been raised about the emergence of breakthrough infections [1,2].

The quantification of breakthrough infection is challenging outside of randomized trials [3]; however, data from surveillance systems have revealed a non-negligible increased rate of breakthrough infection cases across time [4]. The development of a breakthrough COVID-19 infection seems to be linked to several factors, such as the host immunity and clinical features, the rapid waning of antibody response after full vaccination, and the spread of new SARS-CoV-2 Variants of Concern (VoC), such as the currently dominant Omicron, which has the ability to escape the vaccine-induced immunity [3].

The rate of severe-critical COVID-19 breakthrough infections is higher in high-risk populations, such as older, high-comorbid, and immunocompromised patients who may have a tendency to reduced or impaired postvaccine circulating antibodies [5,6]. For these reasons, in parallel to vaccination and other preventive measures, there remains a need for SARS-CoV-2 passive immunotherapy targeting fragile populations in case of breakthrough infection.

The emergency use authorization (EUA) of SARS-CoV-2 anti-spike monoclonal antibodies (mAb) in mild-to-moderate COVID-19 was issued on the basis of randomized placebo-controlled trials (RCT), which have demonstrated a significant reduction of COVID-19 progression and hospitalization [7,8,9,10]. The main limitations of these RCTs, which hinder the applicability of their results in the current clinic and epidemiologic scenario, are the lack of the inclusion of patients with a history of vaccination and of the assessment of infecting VoC. Recent in vitro studies, in fact, have reported a reduction in neutralization activity of some of the currently authorized mAb against Omicron [11,12], with a potential worrisome decrease in their therapeutic efficacy. These evidence gaps are of even greater concern in immunocompromised patients and in those experiencing breakthrough infection, especially when infected with the Omicron variant.

This study aims to assess and compare COVID-19 clinical progression in high-risk outpatients receiving mAb treatment for SARS-CoV-2 primary infection and breakthrough infection, and to identify the predictors of hospitalization, in order to inform clinical decision-making.

## 2. Materials and Methods

### 2.1. Study Design and Population

This is an observational prospective cohort study carried out within the ORCHESTRA (Connecting European Cohorts to Increase Common and Effective Response to SARS-CoV-2 Pandemic) project. The study included all high-risk outpatients aged ≥18 years with microbiologically confirmed SARS-CoV-2 infection, presenting mild-to-moderate COVID-19, who received mAb therapy from 18 March 2021 to 15 February 2022 at the University Hospital Verona, Italy. An ad hoc electronic reporting system was established with general practitioners to improve the recruitment of eligible patients in the Verona province and outskirts. The outpatients were deemed at high risk for COVID-19 progression if at least one of the medical underlying comorbidities was present: (i) obesity, defined as body mass index BMI ≥ 30 kg/m^2^, (ii) any cardiovascular disease (including at least one of the following: arterial hypertension, coronary ischemic disease, congestive heart failure), (iii) any chronic kidney disease requiring/not requiring dialysis, (iv) diabetes mellitus (with or without organ damage), (v) any respiratory disease (including at least one of the following: chronic obstructive pulmonary disease, asthma, pulmonary hypertension, obstructive sleep apnea syndrome, restrictive lung disease), (vi) any neurological disease (including at least one of the following: Alzheimer disease, Parkinson disease, stroke, multiple sclerosis, amyotrophic lateral sclerosis), (vii) any immunocompromising condition (including at least one of the following: active solid neoplasm or onco-hematological disease, solid or bone marrow transplantation, any auto-inflammatory disease, ongoing immunosuppressive therapy).

Outpatients received one of the following mAb regimens: bamlanivimab, bamlanivimab-etesevimab, casirivimab-imdevimab, and sotrovimab. The type of prescribed mAb regimen mainly relied on the regional epidemiological updates on the circulating VoC and on the available supply at the hospital center.

Outcome variables were collected within 28 days by means of a follow-up evaluation in person or by phone, based on the feasibility of the study staff. The study was continued until 13 March 2022, and concluded with the follow-up evaluation of the last included patients.

### 2.2. Variables Description and Definition

The following variables were collected for each patient at the time of study inclusion: age, gender, time from COVID-19 symptoms onset to mAb administration (days), type and number of medical underlying comorbidities, type of SARS-CoV-2 vaccination schedule (manufacturer, number of doses and dates of administration), and type and date of mAb regimen received. Mild-to-moderate COVID-19 was defined by scores 2 (symptomatic, independent) or 3 (symptomatic, assistance needed) of the World Health Organization (WHO) Clinical Progression Score [13]. A breakthrough case was defined as a subject who has a microbiologically documented SARS-CoV-2 infection (detection of SARS-CoV-2 antigen or RNA on the nasopharyngeal swab (NPS) ≥ 14 days after completing the entire primary course of any EMA authorized SARS-CoV-2 vaccine (according to the manufacturer), including the booster dose if the subject was eligible [14]. All patients who did not meet this definition were considered to have a primary SARS-CoV-2 infection. Patients with previous microbiologically documented SARS-CoV-2 infection were excluded from the study. The activity of the prescribed mAb regimen toward the infecting VoC was assessed on the basis of the most recent in vitro data on neutralization activity provided by the National Institute of Health (NIH) COVID-19 treatment guidelines [15], Supplementary Table S1.

All data were collected by dedicated ORCHESTRA personnel and managed through a REDCap secure platform hosted at CINECA Consortium, in accordance with the General Data Protection Regulation (GDPR) EU 2016/679. The study was approved by the Local Ethic Board (Protocol number 19293). All patients signed the informed consent before study inclusion.

### 2.3. Virological and Serological Analyses

At the time of mAb administration, each patient underwent the collection of an NPS for SARS-CoV-2 variant identification and serum samples for anti-SARS-CoV-2 serology quantification. Consensus viral sequences were extracted, clades and lineages assigned using NextStrain (https://nextstrain.org/nextclade/sars-cov-2?branchLabel=aa (accessed on 3 August 2022)) and Phylogenic Assignment of named Global Outbreak LINeages (PANGOLIN) [16]. SARS-CoV-2 anti-Nucleocapsid (anti-NCP), anti- Receptor Binding Domain (RBD), and anti-Spike antibody titers were measured and expressed in Antibody Units (AU)/mL, converted to WHO Binding Antibody Units (BAU)/mL [17]. Serology results were reported quantitatively and qualitatively according to five tiers: negative, inconclusive, low, medium, and high. Details on the applied methodology are reported in Supplementary Table S2.

### 2.4. Outcomes

The primary outcome was hospitalization for COVID-19-related causes within 28 days after mAb infusion. Secondary outcomes were: length of hospital stay, need for non-invasive or mechanical ventilation, and death.

### 2.5. Statistical Analysis

Descriptive analyses were computed on the total population and stratified by type of infection: primary infection versus breakthrough infection. Baseline variables and outcome variables of subjects with or without breakthrough infection were assessed and compared using Chi-square ‘χ2′ for categorical variables and Wilcoxon or Kruskall Wallis tests for continuous variables. Categorical and continuous variables were expressed as frequency and proportions and median and Q1–Q3, respectively. Subgroup analyses on anti-SARS-CoV-2 serology titers in breakthrough infection were compared across three population groups: elderly >75 years, immunocompromised, and immunocompetent ≤75 years. Factors associated with the primary outcome were identified using multivariate logistic regression and results were summarized with adjusted odds ratios (aORs) along with a 95% confidence interval (95%CI). A *p-*value less than 0.05 was regarded as statistically significant. All analyses were conducted with STATA^®^, version 17.0 (StataCorp LP, College Station, TX, USA).

## 3. Results

### 3.1. Description of the Study Population

Overall, 847 mild-to-moderate COVID-19 outpatients were included in the study. Four hundred and twenty-four (50.1%) were males, the median population age was 63 years (Q1–Q3, 52–73). The most represented underlying comorbidities were cardiovascular disease (433 patients, 52.3%), followed by obesity (399 patients, 47.1%), and chronic respiratory disease (149 patients, 17.6%). Four hundred and ten patients (48.4%) suffered from at least two underlying comorbidities. One-hundred and eighty patients (21.3%) had an immunocompromising condition: 85 (47.2%) active tumor (39 onco-hematological disease and 46 solid cancer), 64 patients (35.5%) auto-inflammatory disease, and 27 (15.0%) solid organ transplant. Four patients (2.2%) were HIV-infected subjects. One hundred and seventy-six (98%) patients were being treated with ongoing immunosuppressive therapy. Baseline characteristics of the study population are reported in Table 1.

Four-hundred and fourteen (48.8%) patients were not vaccinated or partly vaccinated for SARS-CoV-2 and developed a primary infection; 433 (51.2%) were fully vaccinated with two or three dosages and developed a breakthrough infection. Among the primary infection group, 370 (89.3%) patients had no history of vaccination and 44 (10.7%) patients received an incomplete vaccination course. Among the breakthrough infection group, 334 (75.3%) were administered a primary vaccination course and had the breakthrough infection diagnosis with a median time of 156 days (Q1–Q3, 123–195) after the last vaccine dose. One hundred and nine patients (24.7%) received a primary vaccination course with the additional booster dose and had a breakthrough infection diagnosis with a median time of 70 days (Q1–Q3, 24–88) after the last vaccine dose. Vaccine details are displayed in Supplementary Table S3.

A different distribution of clinical and demographic characteristics was observed between primary and breakthrough infection groups. Patients with breakthrough infection were likely to be older (66 years, 51–73 versus 60 years, 50–71, *p* < 0.001), and to suffer from an immunocompromising condition more frequently (124, 14.6% patients versus 56, 6.6% patients, *p* < 0.001), compared to the primary infection group, Table 1.

### 3.2. Virological Characteristics

Sequencing was successfully performed in 715 (84.4%) patients. One hundred and fifty-three (21.4%), 399 (55.9%), and 163 (22.7%) patients were infected with Alpha, Delta, and Omicron VoC, respectively. Among Omicron patients, 152 were infected with 21K variant (Pango lineage: BA.1 or BA.1.1 subvariants) and 11 patients with 21L variant (Pango: BA.2 subvariant). The variants distribution across the study period is displayed in Supplementary Figure S1. Alpha was detected exclusively in the primary infection group and Omicron was more frequent in the breakthrough infection group, Table 1. Based on the NIH in vitro data on neutralization activity, 62 patients (38%) infected with Omicron received an in vitro active mAb, Supplementary Table S5.

### 3.3. Clinical Outcomes

The overall 28-day COVID-19-related hospitalization rate was 5.9% (50/847 patients), with a significantly increased rate (*p* < 0.001) in the primary infection group (42/414 patients, 10.1%) compared to the breakthrough infection group (8/433 patients, 1.8%). The length of hospital stay was similar between the two groups, Table 2. Thirty-four (68%) hospitalized patients were older than 65 years and 31 (62%) had at least two underlying comorbidities. Twenty-seven (54%) patients were infected with Alpha, 18 (36%) patients were infected with Delta and five patients (10%) were infected with the Omicron variant. Three patients with Omicron received an in vitro active mAb (sotrovimab). Eleven (22%) patients were immunocompromised, and five (10%) were in the breakthrough infection group. Ventilation support was required in nine patients with primary infection versus one patient with a breakthrough infection. Two patients died, one with primary and one with breakthrough infection. A full description of the characteristics of the hospitalized patients is reported in Supplementary Table S4. Anti-RBD, anti-spike, and anti-NCP antibody measurements according to the variant are shown in Supplementary Table S8.

### 3.4. Predictors for COVID-19 Related Hospitalization

In the bivariate analysis, patients with breakthrough infection (OR 0.11, 95%CI: 0.08–0.35, *p* < 0.001) were less likely to experience 28-day COVID-19-related hospitalization compared to patients with primary infection. Advanced age (OR 1.05, 95%CI: 1.03–1.08, *p* < 0.001), cardiovascular disease (OR 2.22, 95%CI: 1.19–4.13, *p* = 0.012), and male gender (OR 2.01, 95%CI: 1.10–3.68, *p* = 0.022) were significantly associated with the primary outcome, Table 3.

After adjustment, the regression model showed that the breakthrough infection (aOR 0.12, 95%CI: 0.05–0.27, *p* < 0.001) remained significantly associated with decreased odds of 28-day COVID-19-related hospitalization. Immunocompromising condition (aOR 2.35, 95%CI: 1.08–5.08, *p* = 0.003), advanced age (aOR 1.06, 95%CI: 1.03–1.08, *p* < 0.001), and male gender (aOR 1.97, 95%CI: 1.04–3.73, *p* = 0.037) were independently associated with the primary outcome. Receiving an in vitro active mAb was associated with reduced odds of hospitalization, without achieving statistical significance in the multivariate analysis (aOR 0.70, Q1–Q3: 0.35–1.41, *p* = 0.320).

**Figure 1:**
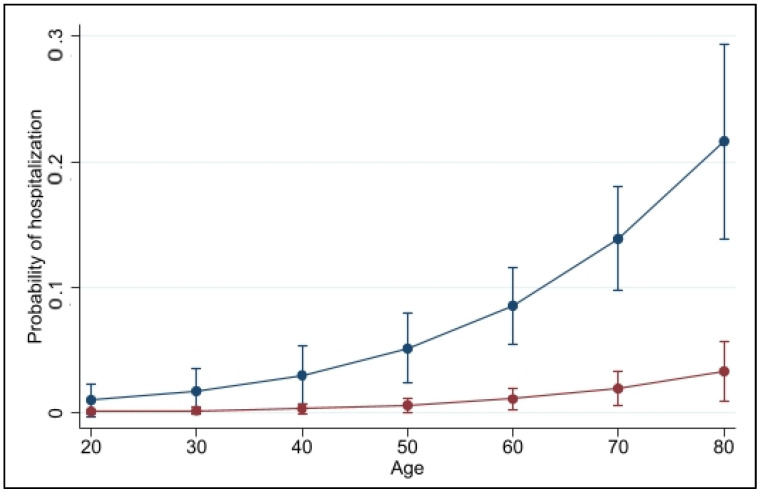
Marginal predicted probabilities of 28-day COVID-19-related hospitalization of outpatients treated with mAb by age and infection type: primary infection (blue curve) and breakthrough infection (red curve).

The marginal predicted probabilities of being hospitalized within 28 days after mAb administration were estimated by type of infection (primary versus breakthrough) and age, Figure 1. The probability of experiencing the outcome increases independently with the increase of age. The curves of the infection types show a lower probability of being hospitalized among patients of younger age while, with age increases, the difference between infection types becomes more substantial. As estimated by the model and confirmed by the respective ORs, the outcome variable is significantly influenced by the infection type, showing a lower probability of hospitalization for the breakthrough infection group compared to the primary infection group.

### 3.5. Serological Characteristics

Anti-SARS-CoV-2 IgGs were successfully quantified in 547 (64.6%) patients. The median SARS-CoV-2 anti-spike antibody titers were significantly higher (*p* < 0.001) in the breakthrough infection group (842, Q1–Q3: 294–1997 BAU/mL) compared to the primary infection group (1.26, Q1–Q3: 0.40–11.18 BAU/mL). The anti-NCP antibodies titers remained low in the study population, with no differences between the two groups, Table 1. Anti-SARS-CoV-2 serology levels according to the detection ranges are reported in Supplementary Table S6.

**Figure 2:**
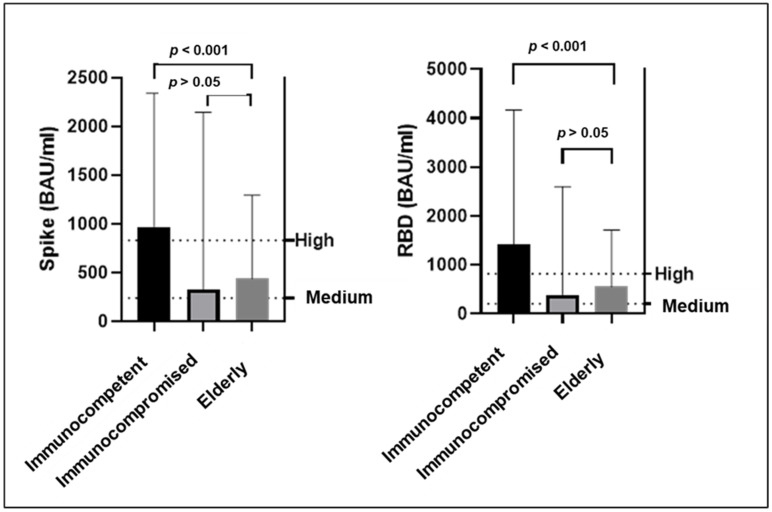
Median SARS-CoV-2 anti-spike and anti-RBD IgGs in immunocompetent, immunocompromised, and elderly patients experiencing a breakthrough infection. Medium and High lines indicate the WHO international standards for anti- SARS-CoV-2 immunoglobulins.

In the breakthrough infection group (n = 278), median SARS-CoV-2 anti-spike and anti-RBD antibody titers were significantly lower (*p* < 0.001) in immunocompromised (anti-spike 327 BAU/mL, Q1–Q3: 50–2094; anti-RBD 387 BAU/mL, Q1–Q3: 23–2591) and elderly (anti-spike 440 BAU/mL, Q1–Q3: 171–1250, anti-RBD 556 BAU/mL, Q1–Q3: 465–3777) groups, compared to the levels in the immunocompetent group (anti-spike 951 BAU/mL, Q1–Q3: 439–2049; anti-RBD 1449 BAU/mL, Q1–Q3: 602–3873) as shown in Figure 2, Supplementary Table S7.

## 4. Discussion

To our knowledge, this is the first study investigating the clinical impact of mAb treatment in outpatients comparing vaccinated versus not vaccinated high-risk patients for severe COVID-19 progression. Compared to patients with primary infection, the hospitalization rate was significantly lower among patients with breakthrough infection and, despite the worse baseline clinical conditions, these patients showed a less severe COVID-19 clinical course. Through a comprehensive assessment of clinical, virological, and serological determinants, the study identified that male patients with immunocompromised status and advanced age have a higher risk for hospitalization, even in case of breakthrough infection, and therefore they could benefit most from early treatment with mAb. For the first time, this study assessed the clinical efficacy of each mAb regimen against the infecting VoC and considered it as a potential outcome predictor.

The efficacy of mAb as an early treatment in preventing disease progression has been widely demonstrated by both randomized and post-clinical trial data; however, all these studies focused exclusively on patients with no history of vaccination [7,8,9,10]. A growing number of observational studies have revealed that patients with breakthrough infection are increasing and may develop severe COVID-19 requiring hospitalization and oxygen supplementation [5,18,19]. In the light of these observations, it is clear that passive immunization remains a key-treatment strategy in breakthrough infection, although data on the clinical role of mAb in fully vaccinated individuals are very limited and conclusions are hard to be drawn [20,21].

Our data revealed that the hospitalization rate was significantly lower in patients with breakthrough infection compared to those with primary infection (10.1% versus 1.8%). Moreover, despite the worse clinical conditions at baseline, these patients had a lower need for ventilatory support compared to patients with primary infection. On the one hand, this result confirms the high efficacy of the currently authorized SARS-CoV-2 vaccination in preventing severe COVID-19 [22,23]. On the other hand, the hospitalization of patients with breakthrough infection occurred irrespective of mAb administration, suggesting that the risk assessment of disease progression, besides the immunity characteristics, might rely depend on several other factors, including host determinants (age, underlying illnesses) and virus profile (variant, pathogenicity) [3].

Our model estimated that patients with immunocompromised conditions had more than a two-fold risk of being hospitalized due to COVID-19, even if they received full vaccination. This result is in line with current literature reporting a weakened vaccine-induced immune response in immunocompromised hosts, especially in transplant recipients and in patients with onco-haematological disease [24,25]. When measuring the immune correlates of protection, our study revealed that both SARS-CoV-2 anti-spike and anti-RBD IgG titers in those patients were significantly lower than those reported in the immunocompetent group, confirming an impairment of the immune response to vaccination [26]. The model also identified age as a significant predictor of poor outcomes. The low titers of anti-spike and anti-RBD antibody titers we found in elderly patients can be attributed to senescence of the immune system, which predisposes to severe COVID-19 and impacts not only innate immunity [27] but also the antibody response induced by vaccination [28].

Preliminary in-vitro studies have shown that the currently authorized mAb might lose efficacy against the currently dominant Omicron VoC [11,12,29]. In accordance with these findings, FDA and AIFA have revised the EUAs for bamlanivimab/etesevimab and casirivimab/imdevimab, halting their use, in line with the NIH COVID-19 Treatment Guidelines Panel [15]. In April 2022, FDA revoked the EUA of sotrovimab in the United States because of the large spread of Omicron BA.2 subvariant, against which this regimen shows decreased in vitro activity [15].

Despite these indications, there remains uncertainty around the real clinical effectiveness of these mAb regimens given the unavailability of pragmatic clinical data. This study offered the unique opportunity to appraise the efficacy of these mAb regimens in patients infected with Omicron in a real-life clinical setting. The knowledge of the infecting variant and the mAb regimen prescribed allowed us to assess the mAb efficacy at the individual-level starting from in vitro data. The multivariate regression model demonstrated a trend toward reduction in hospitalization, although statistical significance was not achieved. These findings highlight again that several different factors may contribute to the disease progression and underline that well-designed randomized trials with adequate sample size linking mAb efficacy and VoC are urgently needed to answer this key-clinical question. The first attempt of assessing the clinical efficacy of different mAb regimens by infecting VoC was shown in MANTICO non-inferiority trial. Exploratory data on 319 patients demonstrated superiority of sotrovimab versus casirivimab-imdevimab and bamlanivimab-etesevimab in reducing the time to recovery in patients infected with Omicron BA.1 and BA.1.1. The trial has been prematurely interrupted because of possible futility reasons and no further valuable clinical conclusions in a proper population sample size could be drawn [30].

The ORCHESTRA cohort has some strengths. First, the study incorporated clinical, serological, and virological characteristics in the assessment of clinical outcomes. Second, the SARS-CoV-2 anti-spike and anti-RBD serology mirror quite reliably the vaccine-induced immunity rather than the natural immunity in course of acute infection, given that the serum collection occurred within five days from symptoms onset. Moreover, SARS-CoV-2 anti-NCP antibodies were negative in the wide majority of patients, indicating that the natural immunity was not yet activated.

The study has some limitations. First, the study was conducted in a regional setting in Italy and the external validity to other health care systems or geographical areas with different epidemiology patterns is unknown. Second, this study did not compare the outcomes with an untreated control population given that our primary purpose was to compare primary and breakthrough infections and to assess factors associated with hospitalization exclusively in mAb-treated patients.

## 5. Conclusions

Our study identified that elderly and immunocompromised patients are at the greatest risk for COVID-19 progression even in case of breakthrough infection, because of a possible impairment of vaccine-induced immunity. These patients may be prioritized for additional vaccination, pre-emptive strategies, or early treatment with mAb. In the light of the new in-vitro evidence, well-conducted randomized studies should be fostered to evaluate the clinical role of mAb regimens against Omicron and potentially new emerging VoC. Prognostic clinical algorithms combining host determinants, and immunological and virological characteristics should be developed and regularly updated with new literature data in order to ensure the appropriate clinical management of early COVID-19 in high-risk categories and optimize the resource allocation.

## Figures and Tables

**Table 1:** Baseline demographic, clinical, and laboratory data of the study population, overall and stratified by type of infection: primary infection and breakthrough infection.

Variables	All Patients (N = 847)	Primary Infection (N = 414)	Breakthrough Infection (N = 433)	*p* Value
**Demographic characteristics**				
**Age**, (years), median (Q1–Q3)	63 (52–73)	60 (50–71)	66 (51–73)	<0.001
**Age Group**, n (%)				
≥65 years	388 (45.8)	156 (18.4)	232 (27.4)	<0.001
**Gender**, n (%)	424 (50.1)	219 (25.9)	205 (24.2)	0.106
Male				
**Body Mass Index** ≥ 30 kg/m^2^	399 (47.1)	233 (53.9)	176 (40.7)	<0.001
**Clinical characteristics**				
**Underlying comorbidity** **Type**, n%				
Chronic kidney disease	53 (6.3)	18 (2.1)	35 (4.1)	0.025
Cardiovascular disease, any	433 (52.3)	209 (50.5)	234 (54.1)	0.300
Hypertension alone	244 (28.8)	112 (27.1)	132 (30.5)	0.270
Diabetes mellitus	120 (14.2)	56 (6.6)	64 (7.6)	0.601
Neurological disease	52 (6.1)	28 (3.3)	24 (2.8)	0.459
Chronic respiratory disease	149 (17.6)	62 (7.3)	87 (10.3)	0.051
Immunocompromising condition	180 (21.3)	56 (6.6)	124 (14.6)	<0.001
**Number**, n (%)				
≥2 underlying comorbidities	410 (48.4)	183 (44.2)	227 (52.4)	0.017
**Time from symptoms onset to infusion** (days), median (Q1–Q3)	3 (2–5)	4 (2–5)	3 (2–4)	0.005
**Type of mAb regimen prescribed**, n (%)				
Bamlanivimab	43 (5.1)	43 (5.1)	-	
Bamlanivimab-etesevimab	384 (45.3)	198 (23.4)	186 (22.0)	<0.001
Casirivimab-imdevimab	315 (37.2)	136 (16.1)	179 (21.1)	
Sotrovimab	105 (12.4)	37 (4.4)	68 (8.0)	
**In-vitro active mAb**, n (%) (n = 715)				
Yes	614 (86.0)	323 (52.6)	291 (47.4)	
No	101 (14.0)	33 (32.7)	68 (67.3)	0.001
**Laboratory data**				
**Viral variant, NextClade, n (%)** (n = 715)				
20I (Alpha)	153 (21.4)	153 (43.0)	0 (0)	
21A, 21I, 21J (Delta)	399 (55.9)	149 (41.9)	250 (69.6)	<0.001
21K, 21L (Omicron)	163 (22.7)	54 (15.1)	109 (30.4)	
**Baseline anti-SARS-CoV-2 serology** (n = 547)				
Anti-spike, median BAU/mL (Q1–Q3)	NA	1.26 (0.40–11.18)	842 (294–1997)	<0.001
Anti-RBD, median BAU/mL (Q1–Q3)	NA	1.55 (0.48–8.19)	1162 (398–3106)	<0.001
Anti-NC, median BAU/mL (Q1–Q3)	NA	1.03 (0.22–5.42)	1.32 (0.10–5.70)	0.06

Abbreviations: mRNA; messenger ribonucleic acid, RBD: Receptor Binding Domain, NC: Nucleocapsid; NA: non-applicable.

**Table 2:** Primary and secondary outcomes in the total population and by infection type.

Clinical Outcomes	All Patients (N = 847)	Primary Infection (N = 414)	Breakthrough Infection (N = 433)	*p* Value
28-day COVID-19-related hospitalization, n (%)	50 (5.9)	42 (10.1)	8 (1.8)	<0.001
Length of stay, median days (Q1–Q3)	9 (6–14)	9 (7–14)	8 (5–12)	0.339
Need for ventilation, n (%)	10 (1.1)	9 (2.1)	1 (0.2)	
Death, n (%)	2 (0.2)	1	1	

**Table 3:** Predictors related to 28-day COVID-19 related hospitalization on the regression model.

28-day COVID-19 Related Hospitalization				
Variable	Bivariate		Multivariate	
	OR (95%CI)	*p-*Value	aOR (95%CI)	*p-*Value
Age §	1.05 (1.03–1.08)	<0.001	1.06 (1.03–1.08)	<0.001
Gender (male)	2.01 (1.10–3.68)	0.022	1.97 (1.04–3.73)	0.037
Underlying comorbidities ≥ 2	1.79 (0.99–3.23)	0.050		
Diabetes	1.16 (0.53–2.54)	0.702		
BMI ≥ 30 kg/m^2^	0.67 (0.37–1.21)	0.186		
Any immunocompromising condition	1.04 (0.52–2.09)	0.894	2.35 (1.08–5.08)	0.003
Cardiovascular disease	2.22 (1.19–4.13)	0.012	1.49 (0.74–2.99)	0.259
Chronic respiratory disease	1.34 (0.67–2.69)	0.840		
Chronic kidney disease	1.33 (0.45–3.83)	0.601		
Time from symptoms onset to mAb infusion	1.02 (0.99–1.03)	0.083		
Breakthrough infection	0.11 (0.08–0.35)	<0.001	0.12 (0.05–0.27)	<0.001
In vitro active mAb	0.86 (0.45–1.66)	0.666	0.70 (0.35–1.41)	0.320

§ age is considered a continuous variable age is treated as a continuous variable and therefore a one unit increase in age correlates with a 6% increase in the odds of being hospitalized.

## Data Availability

The data presented in this study are available on request from the corresponding author.

## Supplementary Materials

The following supporting information can be downloaded at: https://www.mdpi.com/article/10.3390/biomedicines10092063/s1, Table S1: SARS-CoV-2 Variants and Susceptibility to Anti-SARS-CoV-2 Monoclonal Antibodies (https://www.covid19treatmentguidelines.nih.gov/tables/variants-and-susceptibility-to-mabs/ last update 29 April 2022); Table S2: Methodology used for SARS-CoV-2 variant identification (3a), anti-SARS-CoV-2 IgGs measurement (3b); Table S3: Details on vaccination schedule and timing in patients experiencing primary infection and breakthrough infection; Table S4: Clinical and virological characteristics of hospitalizes patients overall and by infection type (primary *versus* breakthrough infection). Table S5: SARS-CoV-2 Variants and Susceptibility to Anti-SARS-CoV-2 Monoclonal Antibodies in the 715 patients with viral variants available; Table S6: SARS-CoV-2 anti-NCP, anti-RBD and anti-spike antibody measurement in a patient with primary infection versus breakthrough infection; Table S7: SARS-CoV-2 anti-NC, anti-RBD and anti-spike antibody measurement in immunocompetent *versus* immunocompromised patients experiencing breakthrough infection; Figure S1: Distribution of viral variants (using NextStrain) across the study period (18 March 2021–15 February 2022).
