# Supplementary material for: Clinical Impact of Monoclonal Antibodies in the Treatment of High-Risk Patients with SARS-CoV-2 Breakthrough Infections: The ORCHESTRA Prospective Cohort Study
Source: Biomedicines. 2022 Aug 24;10(9):2063. doi: 10.3390/biomedicines10092063 (PMC9495697; doi:10.3390/biomedicines10092063)
Supplement: Supplementary file 1 [file biomedicines-10-02063-s001.zip › biomedicines-1838933-supplementary.pdf]

Supplementary material

Clinical impact of monoclonal antibodies in the treatment of high-risk patients with SARS-CoV-2 breakthrough infections: the ORCHESTRA prospective cohort study

Supplementary Table S1……………………………………………………………………………………...2

Supplementary Table S2……………………………………………………………………………………....3

Supplementary Table S3……………………………………………………………………………………....4

Supplementary Table S4……………………………………………………………………………………....5

Supplementary Table S5……………………………………………………………………………………....6

Supplementary Table S6……………………………………………………………………………………....7

Supplementary Table S7……………………………………………………………………………………....8

Supplementary Table S8………………………………………………………………………………………9

Supplementary Figure S1………………………………………………...…………………………………10

Supplementary Table S1. SARS-CoV-2 Variants and Susceptibility to Anti-SARS-CoV-2 Monoclonal Antibodies.

WHO label	Pango Lineage	Notable Mutations	BAMLA plus ETE		CAS plus IMDE		SOTROVIMAB	
			**In Vitro Susceptibility**	**Anticipated Clinical Activity**	**In Vitro Susceptibility**	**Anticipated Clinical Activity**	**In Vitro Susceptibility**	**Anticipated Clinical Activity**
**Alpha**	B.1.1.7	N501Y	No change	Active	No change	Active	No change	Active
**Beta**	B.1.351	K417N, E484K, N501Y	Marked reduction	Unlikely to be active	No change	Active	No change	Active
**Gamma**	P.1	K417T, E484K, N501Y	Marked reduction	Unlikely to be active	No change	Active	No change	Active
**Delta**	B.1.617.2, non-AY.1/ AY.2	L452R, T478K	No change	Active	No change	Active	No change	Active
**Omicron**	B.1.1.529/ BA.1	K417N, N440K, G446S, E484A, Q493R, N501Y	Marked reduction	Unlikely to be active	Marked reduction	Unlikely to be active	No change	Active
**Omicron**	B.1.1.529/ BA.1.1	R346K, K417N, N440K, G446S, E484A, Q493R, N501Y	Marked reduction	Unlikely to be active	Marked reduction	Unlikely to be active	No change	Active
**Omicron**	B.1.1.529/ BA.2	T376A, K417N, N440K, E484A, Q493R, N501Y	Marked reduction	Unlikely to be active	Marked reduction	Unlikely to be active	Marked reduction	Unlikely to be active

Source: NIH COVID-19 guidelines (last update 29 April 2022). Available at: https://www.covid19treatmentguidelines.nih.gov/tables/variants-and-susceptibility-to-mabs/

**Supplementary Table S2. Methodology used for SARS-CoV-2 variant identification (3a), anti-SARS-CoV-2 IgGs measurement (3b).**
**3a.** RNA was extacted using the MagMAX Viral Pathogen II Nucleic acid kit (ThermoFisher) on a KingFisher Flex Purification System according to manufacturer's instructions. Extracted RNA was subjected to Real-Time (RT) Reverse Transcriptase (RT-)qPCR using the TaqPath™ COVID-19 CE-IVD RT-PCR Kit (ThermoFisher) on a QuantStudio™ 5 RT-PCR instrument (384-well block, 5 colors, ThermoFisher) which detects three genes in the SARS-CoV-2-viral genome: S protein, N protein, and ORF1ab genes. In case of positive Real_Time-qPCR (detection of the MS2 phage positive control and at least two gene targets), the extracted RNA was subjected to automated cDNA conversion and multiplexed library preparation using the Illumina COVIDSeq Test kit (Illumina Inc.) on a Zephyr G3 NGS (PerkinElmer) instrument according to manufacturer 's instruction. Pooled libraries were sequenced using the High Output Kit v2 (Illumina Inc.) with a 1.4 nM PhiX Library positive control v3 using a 1% spike-in on a NextSeq 500/550 instrument (Illumina Inc.). Raw sequencing data quality for each sample was assessed using FastQC (https://www.bioinformatics.babraham.ac.uk/projects/fastqc/) followed by quality trimming using a Phred score cutoff of 25 with TrimGalore v. 0.6.7 (https://github.com/FelixKrueger/TrimGalore). Sequencing was considered successful if an estimated genome coverage >100x was obtained. Read mapping was performed against the SARS-CoV-2 genome (GenBank: NC_045512.2) using the CLC Genomics Workbench v.9.5.3 (Qiagen) with a length and a similarity fraction of 0.5 and 0.8, respectively. Consensus sequences were extracted, and lineages assigned using Phylogenic Assignment of named Global Outbreak LINeages (PANGOLIN).

3b.

IgG titres were measured in serum samples using V-PLEX SARS-CoV-2 Panel 6 Kit (IgG) from Meso Scale Discovery (MSD, MD, USA) according to the manufacturer instructions. IgG titres to the following antigens were measured: Nucleocapsid (NCP), Receptor Binding Domain (RBD), Spike, Spike (D614G), Spike (B.1.1.7), Spike (B.1.351) and Spike (P.1).

Measurements were performed in randomized batches. Briefly, 96-well plates were blocked with MSD blocking buffer A for 30 minutes. All plates were then washed three times with PBS-Tween20 (0.05%). Samples were diluted 1:5,000 in Diluent 100 (MSD), loaded on the plates and incubated for two hours, after which the plates were washed three times again. Detection antibody with a sulfo-tag was added and after another one-hour incubation step plates were washed and read with MSD Gold Read Buffer B on the QuickPlex SQ 120 (MSD). Quantitative IgG results were measured in Antibody Units (AU)/mL, converted to WHO Binding Antibody Units (BAU)/mL using a conversion factor provided by MSD. The detection range is described in the table below.

Stratification of quantitative IgG results.

	Negative	Inconclusive	Low	Medium	High	Units
**anti-Spike**	<4.76	4.76 - <53	53 - <241	241 - <832	>832	BAU/mL
**anti-RBD**	<5.58	5.58 - <45	45 - <205	205 - <817	>817	BAU/mL
**anti-N**	<8.20	8.20 - <12	12 - <295	295 - <713	>713	BAU/mL

The upper limit for "Negative" was determined as the average plus one standard deviation of anti-Spike IgG measurements in 50 serum samples collected before 2019. The lower limits for "Low", "Medium" and "High" were based on the BAU/mL concentrations of "Low" (NIBSC code 20/140), "Mid" (NIBSC code 20/148) and "High" (NIBSC code 20/150) WHO International Standards for anti-SARS-CoV-2 immunoglobulins.

Supplementary Table S3. Details on vaccination schedule and timing in patients experiencing primary infection and breakthrough infection.

**Vaccination information in patients with primary infection (N=414)**	
**No history of vaccination,** n (%)	**370 (89.3)**
**Uncompleted vaccination course,** n (%)	**44 (10.7)**
- 1 mRNA-1273 dose	8 (25.0)
- 1 BNT162b2 dose	24 (75.0)
- Ad26.COV2.S single dose < 14 days from last vaccine dose and SARS-COV-2 infection diagnosis	6 (50.0)
- 2 BNT162b2 doses < 14 days from last vaccine dose and SARS-COV-2 infection diagnosis	6 (50.0)
**Vaccination information in patients with breakthrough infection (n=433)**	
**Completed primary vaccination course***, n (%)	**324 (74.8)**
Homologous schedule	319 (98.4)
- 2 mRNA-1273 doses	14 (4.4)
- 2 BNT162b2 doses	205 (64.3)
- Ad26.COV2.S single dose	17 (5.3)
- 2 ChAdOx1-S doses	83 (26.0)
Heterologous schedule	5 (1.6)
- 1 ChAdOx1-S dose + 1 mRNA-1273 dose	1
- 1 ChAdOx1-S dose + 1 BNT162b2 dose	4
**Time to date of the last vaccine dose to breakthrough infection diagnosis,** median days, (Q1-Q3)	156 (123-195)
**Completed primary vaccination course with additional booster dose****	**109 (25.2)**
Homologous schedule	88 (80.7)
- 3 mRNA-1273 doses	3 (3.4)
- 3 BNT162b2 doses	85 (96.6)
Heterologous schedule	21 (19.3)
- 2 mRNA-1273 doses + 1 BNT162b2 dose	4
- 2 BNT162b2 doses + 1 mRNA-1273 dose	7
- 2 ChAdOx1-S doses + 1 BNT162b2 dose	6
- 2 ChAdOx1-S doses + 1 mRNA-1273 dose	4
**Time to date of the last vaccine dose to breakthrough infection diagnosis,** median days (Q1-Q3)	70 (24-88)

*primary vaccination course: heterologous or homologous two-dose primary vaccine course with the following authorised vaccine types: 2 BNT162b2 (Cominraty, Pfizer-Biontech®), mRNA-1273 (Spikevax, Moderna®), ChAdOx1-S (Vaxzevria, Astrazeneca®) OR 1 dose of Ad26.COV2.S (Johnson & Johnson, Jannsen®)
**booster dose: receipt of additional dose after a completed primary vaccination course

Supplementary Table S4: Clinical and virological characteristics of hospitalized patients overall and by infection type (primary *versus* breakthrough infection)

Variable	All population (n=50)	Breakthrough infection (n=8)	Primary infection (n=42)
**Age, (years),** median (Q1-Q3)	69.5 (61-81)	78 (69.5-80.5)	67.5 (60-81)
**≥ 2 underlying comorbidities**, n (%)	31 (62)	8 (100)	23 (54.8)
**Immunocompromising condition** (including active tumor, auto-inflammatory diseases, and transplant), n (%)	11 (22)	5 (62.5)	6 (14.3)
**Viral variant, Next clade**			
- 20I (Alpha)	27 (69.2)	27 (81.8)	0
- 21A, 21I, 21J (Delta)	10 (25.6)	5 (15.1)	5 (83.3)
- 21K, 21L (Omicron)	1 (3)	1 (16.7)	2 (5.1)
**Vaccination schedule,**			
- Completed primary vaccination course without booster, n (%)		6 (75)	NA
- Completed primary vaccination course with booster, n (%)		2 (25)	NA
**Baseline anti-SARS-CoV-2 serology**	**N=35**	**N=5**	**N=30**
**Anti-RBD,** titer N (%)			
- Negative or inconclusive	22 (62.8)	1 (20)	21 (70)
- Low	1 (2.8)	1 (20)	0
- Medium	2 (5.8)	0	2 (6.6)
- High	10 (28.6)	3 (60)	7 (23.4)
**Anti-NCP,** titer N (%)			
- Negative or inconclusive	25 (71.4)	3 (60)	22 (73.3)
- Low	6 (17.2)	2 (40)	4 (13.4)
- Medium	1 (2.8)	0	1 (3.3)
- High	3 (8.6)	0	3 (10)

Supplementary Table S5. SARS-CoV-2 Variants and Susceptibility to Anti-SARS-CoV-2 Monoclonal Antibodies in the 715 patients with viral variant available.

Green: Active. Red: Unlikely to be active.

WHO label (when applicable)	**Viral Variant** Nextstrain Clade	**mAb regimen**			
		**Bamlanivimab**	**Bamlanivimkab/ Etesevimab**	**Casirivimab/ Imdevimab**	**Sotrovimab**
**Alpha**	20I	**43**	**94**	**16**	**0**
**Delta**	21A	**0**	**3**	**1**	**1**
	21I	**0**	**29**	**25**	**1**
	21J	**0**	**188**	**143**	**8**
**Omicron**	21K (Pango lineages BA.1 and BA.1.1)	**0**	**22**	**68**	**62**
	21L (Pango lineage BA.2)	**0**	**0**	**1**	**10**

Supplementary Table S6. SARS-CoV-2 anti-NCP, anti-RBD and anti-spike antibody measurement in patient with primary infection *versus* breakthrough infection.

Titer N (%)	Anti-spike (n=547)		Anti-RBD (n=547)		Anti-NCP (n=547)	
	**Primary (n=269)**	**Breakthrough (n=278)**	**Primary (n=269)**	**Breakthrough (n=278)**	**Primary (n=269)**	**Breakthrough (n=278)**
**Negative**	170 (63.2)	6 (2.1)	187 (69.5)	8 (2.9)	219 (81.4)	223 (80.2)
**Inconclusive**	53 (19.7)	15 (5.5)	42 (15.6)	11 (3.9)	10 (3.7)	10 (3.6)
**Low**	17 (6.4)	41 (14.7)	15 (5.6)	25 (9.0)	30 (11.2)	45 (16.2)
**Medium**	9 (3.3)	77 (27.7)	8 (3.0)	76 (27.3)	4 (1.5)	0 (0)
**High**	20 (7.4)	139 (50.0)	17 (6.3)	158 (56.9)	6 (2.2)	0 (0)
Abbreviation: RBD: receptor binding domain, NCP: nucleocapsid.						

Supplementary Table S7. SARS-CoV-2 anti-NCP, anti-RBD and anti-spike antibody measurement in immunocompetent *versus* immunocompromised patients experiencing breakthrough infection.

Titer N (%)	Anti-spike (n=278)			Anti-RBD (n=278)			Anti-NCP (n=278)		
	Immuno-competent (n=190)	Immuno-compromised (n=40)	Elderly (n=48)	Immuno-competent (n=190)	Immuno-compromised (n=40)	Elderly (n=48)	Immuno-competent (n=190)	Immuno-compromised (n=40)	Elderly (n=48)
**Negative**	1 (0.5)	5 (12.5)	0 (0)	1 (0.5)	7 (17.5)	0 (0)	152 (80.0)	32 (80)	39 (81.3)
**Inconclusive**	5 (2.6)	5 (12.5)	5 (10.4)	3 (1.58)	4 (10.0)	4 (8.3)	8 (4.2)	0 (0)	2 (4.2)
**Low**	22 (11.6)	5 (12.5)	14 (29.2)	12 (6.3)	4 (10.0)	9 (18.8)	30 (15.8)	8 (20)	7 (14.5)
**Medium**	56 (29.5)	11 (27.5)	10 (20.8)	48 (25.3)	11 (27.5)	17 (35.4)	0 (0)	0 (0)	0 (0)
**High**	106 (55.8)	14 (35)	19 (39.6)	126 (66.3)	14 (35)	18 (37.5)	0 (0)	0 (0)	0 (0)

Abbreviation: RBD: receptor binding domain, NCP: nucleocapsid.

Supplementary Table S8. SARS-CoV-2 anti-NCP, anti-RBD and anti-spike antibody measurement according to the VoC.

Titer N (%)	Anti-spike (n=243)		Anti-RBD (n=243)		Anti-NCP (n=243)	
	**Delta (n=230)**	**Omicron (n=13)**	**Delta (n=230)**	**Omicron (n=13)**	**Delta (n=230)**	**Omicron (n=13)**
**Negative**	5 (2.2)	0 (0)	6 (2.6)	0 (0)	190 (82.6)	8 (61.5)
**Inconclusive**	10 (4.3)	0 (0)	7 (3.1)	0 (0)	7 (3.1)	1 (7.7)
**Low**	38 (16.5)	0 (0)	23 (10)	0 (0)	33 (14.3)	4 (30.8)
**Medium**	67 (29.2)	5 (38.5)	68 (29.5)	3 (23)	0 (0)	0 (0)
**High**	110 (47.8)	8 (61.5)	126 (54.8)	10 (77)	0 (0)	0 (0)
Abbreviation: RBD: receptor binding domain, NCP: nucleocapsid.						

Supplementary Figure S1: Distribution of viral variants (using NextStrain) across the study period (18th March 2021- 15th February 2022).

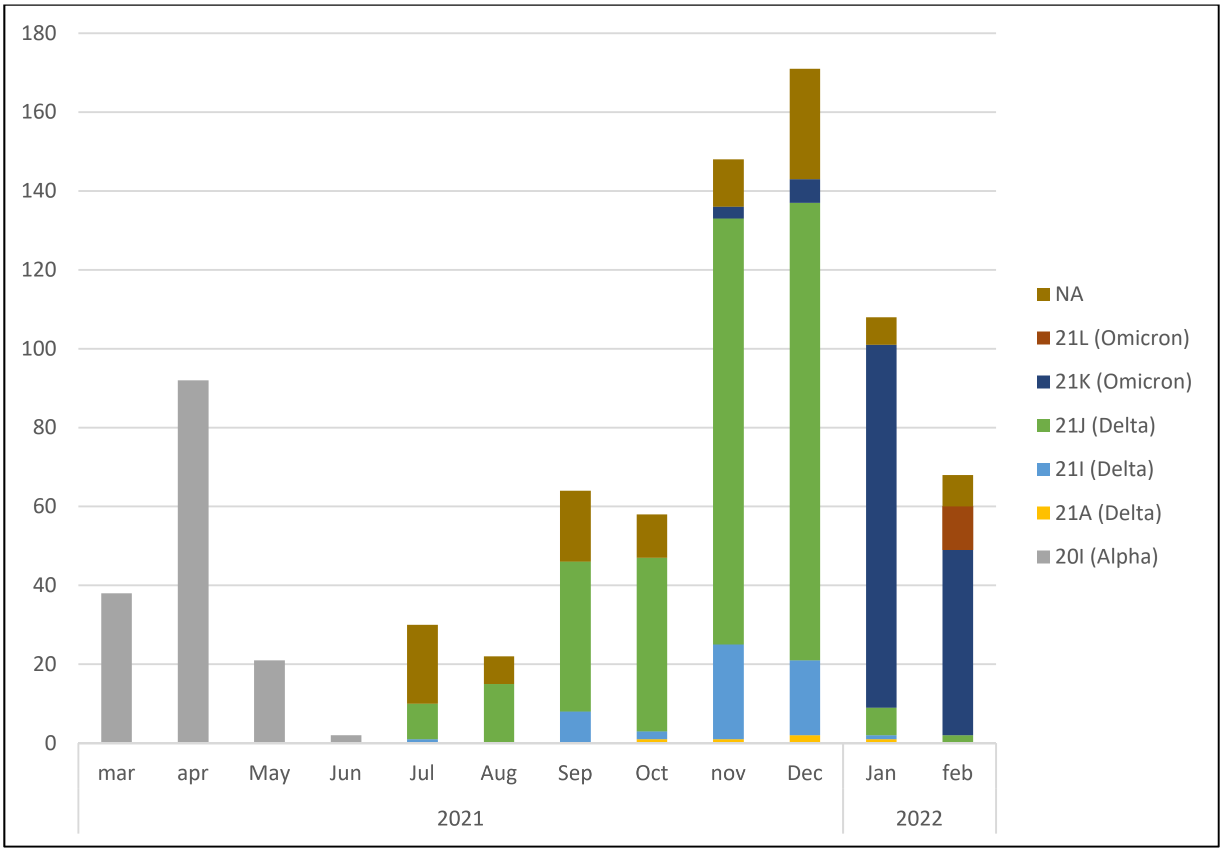

NA: non-available or failed sequencing
